# Identifying the mechanism underlying treatment failure for *Salmonella* Paratyphi A infection using next-generation sequencing – a case report

**DOI:** 10.1186/s12879-019-3821-x

**Published:** 2019-02-26

**Authors:** Hye-Ran Park, Dong-Min Kim, Na-Ra Yun, Choon-Mee Kim

**Affiliations:** 1 Department of Internal Medicine, College of Medicine, Chosun University, 588 Seosuk-dong, Dong-gu, Gwangju, 501-717 Republic of Korea; 2 Department of Premedical Science, College of Medicine, Chosun University, Gwangju, Republic of Korea

**Keywords:** *Salmonella enterica* Paratyphi A, Osteomyelitis, Next-generation sequencing, Antibiotic resistance

## Abstract

**Background:**

*Salmonella* is a notorious pathogen that causes gastroenteritis in humans and the emergence of resistance to third-generation cephalosporins and azithromycin have raised concern. There has been rare case of *Salmonella* Paratyphi A infection accompanied by spondylitis. Here, we report a case of initial antibiotic treatment failure in a Korean man with *Salmonella* Paratyphi A infection and conducted next-generation sequencing (NGS) to determine the cause of failure of initial treatment for *Salmonella* Paratyphi A infection.

**Case presentation:**

A 70-year-old man was admitted to Chosun University Hospital with reported consistent low back pain with a history of having 5 days of chills and fever in another hospital a month ago. He was administered ceftriaxone (2 g daily) for 18 days including initial treatment to cover *Salmonella enterica.* The antimicrobial susceptibility test using MIC plate, found that the identified organism was resistant to ciprofloxacin and nalidixic acid. Moreover, the *Salmonella* Paratyphi A isolates were found to have an MIC > 16 mg/L for azithromycin, as he had resistance to both azithromycin and nalidixic acid, the treatment was switched to a combination of ciprofloxacin and cefotaxime. We carried out next-generation sequencing (NGS) to determine the cause of failure of initial treatment for *Salmonella* Paratyphi A infection. NGS showed that the amino acid substitution GyrA S83F and the expression of multiple RNA-family efflux pumps led to a high-level resistance to quinolone. No genes related to ceftriaxone resistance, such as *CTX-M*, *CMY-2*, or other extended-spectrum beta-lactamases were identified in *Salmonella enterica* Paratyphi A using NGS. The GyrA S83F mutation and the expression of multiple RNA-family efflux pumps may have contributed to the treatment failure of ceftriaxone, even though the MIC of the isolate to ceftriaxone was less than 1.

**Conclusion:**

This case involved a *Salmonella* Paratyphi A infection accompanied by spondylitis. To our knowledge, this is the first report to elucidate the mechanism underlying antimicrobial resistance using NGS.

## Background

*Salmonella* is a notorious pathogen that causes gastroenteritis in humans, and 94 million cases of salmonellosis are reported globally every year [1]. Infections are systemic, characterized by fever and gastrointestinal symptoms, and are associated with significant morbidity [2]. Death can occur, especially if appropriate antimicrobial therapy is delayed [3]. Fluoroquinolones became the first-line antimicrobial therapy following their introduction in the 1980s, and they were initially associated with rapid fever clearance and low rates of both relapse and chronic faecal carriage [4]. However, reduced ciprofloxacin susceptibility (MIC 0.06–0.25 mg/L) has become increasingly prevalent in *Salmonella enterica* Typhi and *Salmonella enterica* Paratyphi A, and has been associated with clinical failure [5, 6]. Fluoroquinolone-resistant strains of *Salmonella* Typhi and *Salmonella* Paratyphi A have recently emerged in tropical and subtropical regions of the world, such as southeast Asia and Africa [7]. In the UK, > 90% of *Salmonella* Typhi and *Salmonella* Paratyphi A isolates acquired from India between 2006 and 2007 were found to be nalidixic acid-resistant [8]. Alternative antimicrobials, including third-generation cephalosporins and azithromycin, are now being increasingly used as first-line therapies [9]. Reports of emergence of resistance to third-generation cephalosporins and azithromycin have raised concern amongst clinicians [10, 11]. A case of clinical failure under azithromycin treatment in a case of bacteremia due to *Salmonella enterica* Paratyphi A was reported in 2014 [7].

Here, we report a case of initial antibiotic treatment failure in a Korean man with *Salmonella* Paratyphi A infection and conducted next-generation sequencing (NGS) to determine the cause of failure of initial treatment for *Salmonella* Paratyphi A infection.

## Case presentation

**Fig. 1:**
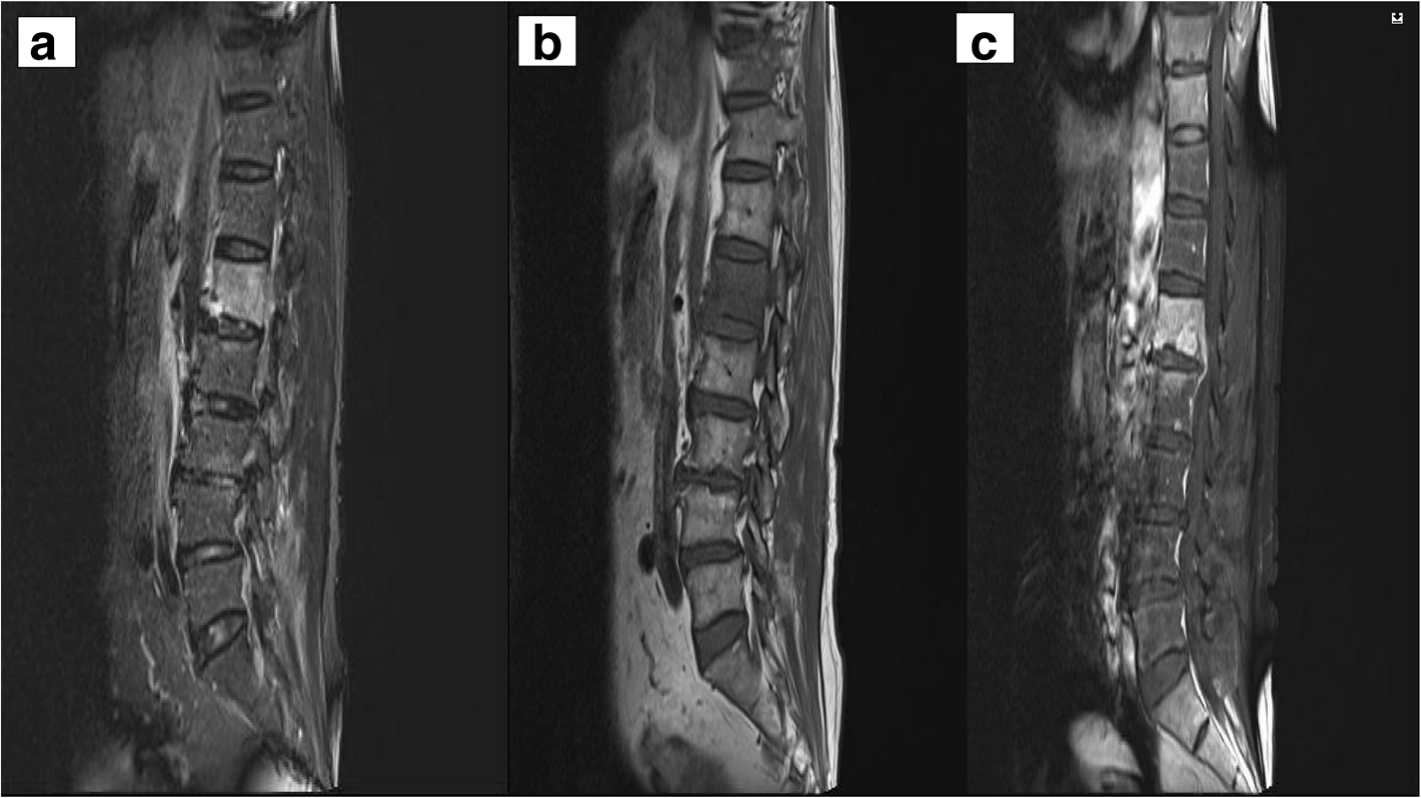
**a** L-spine T2_tse_sagittal, **b** L-spine T1_tse_sagittal, and **c** L-spine T1_tse_sagittal_fat suppressed. MRI of the lumbosacral spine shows altered signals in the L1 vertebral body (in the local clinic on 30 December 2017). MRI revealed whole bone marrow oedema with endplate lytic changes in the L1 body and focal marrow oedema in the upper endplate of L2 bodies. Additionally, it showed mild destruction of intervertebral disc at L1–2. Adjacent paravertebral soft tissue thickening and small abscess in left psoas muscle were found. These findings are considered to be indicative of pyogenic spondylitis

In January 2018, a 70-year-old man residing in South Korea was admitted to Chosun University Hospital with reported consistent low back pain. At first, he had been admitted to a local hospital on 24 November 2017, a month before visiting Chosun University Hospital with a history of 5 days of chills and fever. In the local hospital, in view of the possibility of acute pyelonephritis, he was first treated with intravenous ceftriaxone at a dosage of 2 g daily. Two days after admission, back pain started. During antibiotic treatment, blood cultures taken on admission yielded *Salmonella enterica*. He remained on ceftriaxone (2 g daily) for 18 days including initial treatment to cover *S. enterica*. Upon follow-up blood culture, no bacteria were detected on the 8th and 25th days after starting treatment, and the patient no longer had fever; he was subsequently discharged from the local hospital on 19 December 2017. However, he consistently suffered from lower back pain, nausea, and vomiting; he was re-admitted to the same local hospital 9 days after his discharge. When he was re-admitted to the local hospital again on 30 December 2017, magnetic resonance imaging (MRI) was performed and L1 spondylitis was demonstrated. MRI revealed whole bone marrow oedema with endplate lytic changes in the L1 body and focal marrow oedema in the upper endplate of L2 bodies. Additionally, mild destruction of intervertebral disc at L1–2 was shown. These findings were considered to be indicative of pyogenic spondylitis (Fig. 1a, b, c). He was empirically treated with cefazolin (1 g, 3 times a day) for 10 days to cover the possibility of *Staphylococcus aureus* infection, which is a common cause of pyogenic spondylitis. Then, blood cultures were tested and yielded *S. enterica* again. Finally, he was transferred to Chosun University Hospital, and bone biopsy of L spine was performed on 3 January 2018. He had no fever, and the initial blood test was generally unremarkable except that his erythrocyte sedimentation rate (ESR) level was 108 mm/h. After 7 days, the biopsy results of bone and blood cultures were positive for *Salmonella enterica*. Cultures of bone and blood obtained during biopsy at the Chosun University Hospital grew O:2-positive *Salmonella*. The organism was serotyped to be serovar Paratyphi A ([1],2,12:a:-) by the tube agglutination method combining *Salmonella* O and H (flagella) antigens according to the Antigenic Formulae of the *Salmonella* serovars.

After the bacteria were identified, with suspicions of metastatic spondylitis, he was treated with ciprofloxacin for 13 days. Computed tomography (CT) was performed for further evaluation. CT scans revealed L1–2 spondylitis spreading to the left psoas muscle. Fluorodeoxyglucose positron emission tomography (FDG PET) was performed to detect clinically undetected diseases in different sites, and it showed hypermetabolism and bone destruction in L1 and L2 vertebral bodies, as well as mild hypermetabolism in the right facet joint between L4 and L5 vertebral bodies. The antimicrobial susceptibility test using MIC plate by the Chosun University Hospital and Health and Environment Research Institute of Gwangju City found that the identified organism was resistant to ciprofloxacin and nalidixic acid (Table 1). Since susceptibility tests for macrolides could not be carried out due to unavailability of the disc, the specimen was sent for testing to Korea Centers for Disease Control and Prevention. While waiting for results, the antibiotic used in the treatment of the patient was changed from ciprofloxacin to azithromycin due to the identified resistance to ciprofloxacin and nalidixic acid. While he was treated with azithromycin for 15 days, C-reactive ptrotein (CRP) gradually decreased to the normal value of 0.09 mg/dL; ESR level decreased as well, but not to normal levels (CRP: 0.09 mg/dL, ESR: 44 mm/h on the 20th day of admission). He was discharged and followed up through an outpatient clinic. Seven days later, the *Salmonella* Paratyphi A isolates were found to have an MIC > 16 mg/L for azithromycin. As he had resistance to both azithromycin and nalidixic acid, the treatment was switched to a combination of ciprofloxacin and cefotaxime. While he was treated with combination therapy for 2 months, his clinical symptoms such as back pain reduced, and the ESR level gradually decreased to normal (ESR: 20 mm/h on the 64th day of combination therapy).

**Table 1:** Antimicrobial susceptibility test using MIC plate performed in Chosun University Hospital and Health and Environmental Research Institure of Gwangju City

No.	Sample	AN	AM	CZ	CTX	CAZ	TGC	GM	TZP	AMC	AZM	FEP	FOX	ETP	CIP	IPM	SXT	NAL
2017-12-30	blood	<=2	4	<=4	<=1	<=1	1	<=1	<=4	<=2	<=1	<=1	<=4	<=0.5	1	<=0.25	<=20	128<
	interpretations	R	S	R	S	S	S	R	S	S	S	S	R	S	R	S	S	R
2018-01-03	bone Biopsy	<=2	4	<=4	<=1	<=1	2	<=1	<=4	<=2	<=1	<=1	<=4	<=0.5	1	<=0.25	<=20	128<
	interpretations	R	S	R	S	S	S	R	S	S	S	S	R	S	R	S	S	R

Nalidixic acid was tested at the Health and Environment Research Institute, and the remaining interpretations were obtained at Chosun University Hospital

*AN* Amikacin, *AM* Ampicillin, *CZ* Cefazolin, *CTX* Cefotaxime, *CAZ* Ceftazidime, *TGC* Tigecycline, *GM* Gentamicin, *TZP* Piperacillin/Tazobactam, *AMC* Amoxicillin/ClavulanicAcid, *AZM* Aztreonam, *FEP* Cefepime, *FOX* Cefoxitin, *ETP* Ertapenem, *CIP* Ciprofloxacin, *IPM* Imipenem, *SXT* Trimethoprim/Sulfamethoxazole, *NAL* Nalidixic acid

We carried out NGS to determine the cause of failure of the initial treatment for *Salmonella* Paratyphi A infection and elucidate the mechanism underlying antimicrobial resistance in *Salmonella* Paratyphi A.

Purified genomic DNA was randomly sheared to yield DNA fragments approximately 350 bp in size using a Covaris S2 Ultrasonicator. Library preparation was performed using the Illumina TruSeq DNA PCR-free Preparation Kit following the manufacturer’s instructions. Adaptor enrichments were performed using PCR according to the manufacturer’s instructions. The final library size and quality were evaluated electrophoretically with an Agilent High Sensitivity DNA Kit. The 100-bp paired-end reads were sequenced on the Illumina HiSeq 2500 platform. Further image analysis and base calling were performed with RTA 2.7.3 (Real Time Analysis) and bcl2fastq v2.17.1.14.

The draft genome was assembled using A5 pipeline ver. 20,160,825 with paired-end reads. For annotation of the prokaryotic genome, Prokka v1.1.0 was used. The gene models were predicted through the open reading frame (ORF) finding method using prodigal v2.6.2. Then, tRNA, rRNA, and repetitive sequences were identified using Aragon v1.2.36, barrnap v0.6, and minced v0.2.0, respectively. For functional annotation, genes were searched against the UniProt and NCBI RefSeq databases using BLASTP v2.2.29+ with an *E*-cutoff value of 1 × 10^− 6^. Protein domains were also searched against Pfam using HMMER 3.1b1.

Resistance genes were identified using Resistance Gene Identifier (RGI) in the Comprehensive Antibiotic Resistance Database (CARD, http://arpcard.mcmaster.ca) using predicted peptide sequences. All sequences were run through all databases in CARD with a selected threshold of ID = 98.00%.

The clinical isolates were identified with a VITEK II automated system (bioMérieux, Marcy-l’Etoile, France). Tests for antimicrobial susceptibility including MIC were performed with the VITEK II system. In Chosun University Hospital, we cultured the blood collected on 30 December 2017 in the local hospital and conducted antimicrobial susceptibility tests using Clinical and Laboratory Standards Institute (CLSI) guideline (Table 1).

The antimicrobial susceptibility test performed by the Health and Environment Research Institute of Gwangju City showed that the identified organism from the closed pus taken from bone biopsy when the patient was admitted to Chosun University Hospital on 3 January 2018 exhibited resistance to ciprofloxacin (MIC = 1 mg/L) and nalidixic acid (MIC> 128 mg/L) (Table 1). The antibiotic resistance test results for the blood samples obtained on December 30, 2017 and the biopsy samples obtained on January 3, 2018 were the same. The isolate also presented resistance to macrolides (MIC> 16 mg/L) in a test performed by the Korea Centers for Disease Control and Prevention. We carried out NGS to determine the cause of failure of the initial treatment for *Salmonella* Paratyphi A infection and elucidate the mechanism underlying antimicrobial resistance in *Salmonella* Paratyphi A. The isolate used for NGS was taken in the local hospital when he was readmitted in 30 December 2017.

Next-generation sequencing results for *Salmonella* Paratyphi A with a selected threshold of ID = 98.00% showed that there are antimicrobial resistance gene families present in the isolate, including resistance-nodulation-cell division (RND) antibiotics efflux pumps such as mdsC, CRP, and sdiA; gyrA associated with fluoroquinolone resistance such as *Salmonella enterica* gyrA; and MATE transporters such as MdtK and AAC (6′)-ly (Table 2). Based on the results, the most likely cause of treatment failure was a RND antibiotic efflux pump. Meanwhile azithromycin resistance genes such as *mph* and *mef* were not identified (Table 2). There were also no resistance genes related to ceftriaxone, such as *CTX-M*, *CMY-2*, or other extended-spectrum beta-lactamases.

**Table 2:** NGS (next-generation sequencing) of *Salmonella* Paratyphi

Best_Hit_ARO	Drug class	Best_Identities	AMR gene family	SNPs_in_Best_Hit_ARO		
*Salmonella enterica* gyrA conferring resistance to fluoroquinolones	nybomycin; fluoroquinolone antibiotic	99.89	fluoroquinolone resistant gyrA	S83F		
mdsC	carbapenem; monobactam; cephamycin; penam; penem ;phenicol antibiotic; cephalosporin	100	RND antibiotic efflux pump			
CRP	macrolide antibiotic; fluoroquinolone antibiotic; penam	98.57	RND antibiotic efflux pump			
MdtK	fluoroquinolone antibiotic	98.95	MATE transporter			
AAC(6′)-Iy	aminoglycoside antibiotic	98.62	AAC(6′)			
sdiA	triclosan; glycylcycline; rifamycin antibiotic; phenicol antibiotic ;fluoroquinolone antibiotic; cephalosporin; tetracycline antibiotic; penam	99.17	RND antibiotic efflux pump			

All sequences were run out through all databases in ResFinder with a selected threshold of ID = 98.00%

*RND* resistance-nodulation-cell division, *ABC* ATP-binding cassette, *MFS* major facilitator superfamily, *MATE* multidrug and toxic compound extrusion

## Discussion and conclusion

In 2008, a case of L3/4 vertebral osteomyelitis due to *Salmonella* Paratyphi A was first reported with bacteriological confirmation in Dubai, United Arab Emirates [12]; however, it was not described if the isolate exhibited resistance to nalidixic acid and macrolides.

In this case, we carried out NGS to elucidate the mechanism underlying antibiotic resistance in *Salmonella* Paratyphi A due to the emerging development of osteomyelitis during intravenous ceftriaxone treatment. As of late, there have been no studies involving the use of NGS in identifying the mechanism underlying treatment failure for *Salmonella* Paratyphi A.

A recent study reported that 40% of *Salmonella* isolates in Chennai, India have an MIC of > 0.5 uL/mL against ceftriaxone [13].

There is a wide variety of serotypes and susceptibility results among *Salmonella* spp. isolated from clinical specimens in Korea [14]. The three most common *Salmonella* serotypes are Enteritidis, Typhimurium, and Infantis. These *Salmonella* strains had resistance rates of 38.7% to ampicillin, 23.0% to chloramphenicol, 8.2% to cefotaxime, 8.6% to ceftriaxone, and 6.3% to trimethoprim-sulfamethoxazole [14]. Another study showed the same result, where the major serotypes isolated in Jeollanam-do, Korea, were *Salmonella* Enteritidis and *Salmonella* Typhimurium, where a total of 22 different serotypes were identified, and the major serotypes were *Salmonella* Enteritidis (116 strains, 42.0%) and *Salmonella* Typhimurium (60 strains, 21.7%). The highest resistance was observed in response to nalidixic acid (43.4%), followed by ampicillin (40.5%) and tetracycline (31.6%) [15]. Resistance to nalidixic acid was detected in 81.0% of *Salmonella* Enteritidis isolates. Multidrug resistance was detected in 43.3% of *Salmonella* spp. *Salmonella* Enteritidis and *Salmonella* Typhimurium presented the highest resistance (98.3%) and multidrug resistance (73.3%) rates, respectively [15]. A recent report similar to our case showed that a patient infected with *Salmonella* Paratyphi A was treated with ceftriaxone, but his symptoms remained; therefore, his treatment was changed to azithromycin [7]. Even though the MIC of azithromycin was not elevated (8 mg/L), azithromycin treatment failed. The European Committee on Antimicrobial Susceptibility Testing (EUCAST) states that the wild-type isolates of *Salmonella* Typhi have an MIC ≤16 mg/L. In our case, the MIC of the isolate showed that it was azithromycin-resistant (MIC > 16 mg/L) (Table 2).

Based on our NGS results, azithromycin resistance genes such as *mph* and *mef* were not found; however, it was suggested that the mechanism of resistance to azithromycin was due to a RND antibiotic efflux pump.

In our previous study, a combination of ciprofloxacin and cefotaxime showed synergistic effects against nalidixic acid-resistant *Salmonella* Paratyphi A and B. This combination appears to be more effective than monotherapy and may help reduce the chances that fluoroquinolone-resistant mutants will emerge in patients with severe typhoid fever [16, 17]. In this case, we treated with ciprofloxacin and cefotaxime, and the patient’s clinical features including back pain and ESR level decreased.

Olaquindox-resistant isolates were found to contain the gene combination oqxAB, which encodes an RND family efflux pump, confers resistance to olaquindox quinolones and chloramphenicol, and reduces susceptibility to other antibiotics [18]. OqxAB, a plasmid-mediated RND efflux pump conferring resistance to multiple antibiotics, was found in *Salmonella* isolates recovered from food samples. The overall OqxAB-positive rate of *Salmonella typhimurium* strains was 29% (159 out of 546 isolates), and the yearly rates were 0, 13, 26, 32, 36, 39, and 42% during the years 2005 to 2011, respectively. OqxAB was also found to be associated with multidrug resistance in *S. typhimurium* isolates from Hong Kong and from the Infectious Disease Prevention and Control, National Institute for Communicable Disease Control and Prevention (ICDC), Chinese Center for Disease Control and Prevention, Beijing, China. Among the *S.typhimurium* isolates of the OqxAB-positive group, 94% (Hong Kong) and 98% (ICDC) were resistant to ciprofloxacin (MIC = 2 mg/L); the corresponding resistance rate in the OqxAB-negative *S. typhimurium* isolates from Hong Kong and ICDC was only 11% [19].

Our NGS study showed that expression of multiple RND family efflux pumps such as MdsC, CRP, and SdiA, which may be related to quinolone resistance, may have been responsible for the failure of ceftriaxone treatment (Table 2). The upregulation of endogenous SdeXY-HasF-mediated efflux has been reported to be associated with tigecycline resistance in *Serratia marcescens*, along with increases in MIC for tetracycline, ciprofloxacin, and cefpirome [20]. The overexpression of the BmeB efflux pump has also been reported to cause low-to-intermediate-level clinically relevant fluoroquinolone resistance and can be coupled with GyrA substitutions to cause high-level fluoroquinolone resistance. Finally, it also contributes to high-level clinically relevant resistance to beta-lactams [21]. Our case also showed that both the amino acid substitution GyrA S83F and the expression of multiple RND family efflux pumps led to high-level resistance to quinolone. No resistance genes in *Salmonella* Paratyphi A related to ceftriaxone, such as *CTX-M*, *CMY-2*, or other extended-spectrum beta-lactamases were identified using NGS. However, there was no response or even progression to vertebral osteomyelitis to treatment with third-generation cephalosporins after 18 days of the initial treatment. The GyrA S83F substitution and the expression of multiple RND family efflux pumps may have contributed to the failure of treatment with ceftriaxone, even though the MIC of the isolate to ceftriaxone was less than 1. In our case, there was a possibility that the early onset of metastatic spondylitis accounted for the failure of treatment with a third-generation cephalosporin because the treatment duration was not long enough. However, further studies on RND antibiotic efflux pumps are necessary to truly identify it as the cause of third-generation cephalosporin treatment failure.

In conclusion, this case involved a *Salmonella* Paratyphi A infection accompanied by spondylitis. To our knowledge, this is the first report to elucidate the mechanism underlying antimicrobial resistance using NGS.

## Abbreviations

NGS: Next-generation sequencing
ORF: Open reading frame
PCR: Polymerase chain reaction
RGI: Resistance Gene Identifier
